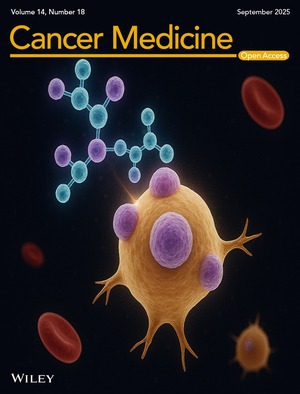# Cover Image

**DOI:** 10.1002/cam4.71245

**Published:** 2025-09-22

**Authors:** Marcela Espinoza, Jorge Rojas‐Vallejos, Nicolás Rodríguez, Gonzalo Guerrero, Miguel López, Natalia Aranguiz, Guillermo Conte, Francisco Samaniego, Nicolás Quinteros, Daniel Astete, Lucas Carcamo, Constanza Flores, Ximena Huerta, Mauricio Chandía, Jorge Valenzuela, Marcelo Navarrete, Yorman Flores, Agatha Larrazabal, Edgar Zapata, Joaquín Jerez

## Abstract

The cover image is based on the article *Real‐World Data on Inotuzumab Ozogamicin for Adult Patients With Relapsed/Refractory Acute Lymphoblastic Leukemia: A GRELAL‐Chile Study* by Marcela Espinoza et al., https://doi.org/10.1002/cam4.71230.